# Comparing IL-6 and IL-10 Serum Levels and Changes Using Propofol and Remimazolam: A Prospective Randomized Controlled Study

**DOI:** 10.7759/cureus.109249

**Published:** 2026-05-20

**Authors:** Donghyo Kim, Hyoungoh Yang, Jihyun An

**Affiliations:** 1 Anesthesiology, Daegu Fatima Hospital, Daegu, KOR

**Keywords:** anesthetics, benzodiazepine, cytokines, hemodynamics, interleukin-10, interleukin-6, intravenous, propofol, sirs

## Abstract

Background: Surgical stress alters cytokine concentrations, triggering systemic inflammatory responses and immune defense mechanisms. Interleukin-6 (IL-6) and interleukin-10 (IL-10) play important roles in perioperative inflammatory responses. General anesthetics have been shown to affect immune function during surgery. Propofol modulates inflammation by decreasing IL-6, IL-10, and tumor necrosis factor-alpha (TNF-α) levels. Remimazolam, a new ultra-short-acting benzodiazepine, is increasingly used as an induction and maintenance anesthetic agent. However, the effects of remimazolam on inflammatory cytokines and perioperative hemodynamic variables remain unclear.

Methods: This prospective randomized study included 46 patients undergoing total laparoscopic hysterectomy or laparoscopic myomectomy under total intravenous anesthesia. Blood samples were collected at four time points: before anesthesia (T0), at the end of surgery (T1), 1.5 h after surgery (T2), and 24 h after surgery (T3). Serum IL-6, IL-10, and TNF-α levels were measured using enzyme-linked immunosorbent assays. However, TNF-α values were below the detectable range of the assay and were therefore excluded from meaningful statistical analysis.

Results: No significant intergroup differences were observed in IL-6 or IL-10 levels (F(2.10, 90.08) = 2.43, P = 0.092; F(1.277, 54.905) = 2.44, P = 0.117) or the IL-6/IL-10 ratio (F(1.874, 80.586) = 0.46, P = 0.620). Compared with the propofol group, the remimazolam group demonstrated significantly higher intraoperative bispectral index (BIS), heart rate, systolic blood pressure, diastolic blood pressure, and mean blood pressure values. Postoperative oxycodone requirements in the post-anesthesia care unit were significantly higher in the propofol group. TNF-α levels remained below the detectable range of the assay and could not be meaningfully analyzed.

Conclusions: No significant intergroup differences were observed in perioperative IL-6 and IL-10 responses between remimazolam and propofol. However, because anesthetic depth may not have been fully equivalent between groups, as reflected by higher BIS values in the remimazolam group, the cytokine and hemodynamic findings should be interpreted cautiously. The absence of statistically significant differences should not be interpreted as evidence of immunologic equivalence between the two anesthetic agents. Further large-scale studies with standardized anesthetic depth are required to clarify the immunomodulatory and hemodynamic effects of remimazolam.

## Introduction

Cytokines are central to the trauma- or infection-induced acute inflammatory response [1]. After major abdominal surgery, changes in cytokine concentration result in the postoperative systemic inflammatory response syndrome (SIRS). Stress hormones, such as glucocorticoids or catecholamines, which are secreted following surgical tissue damage, simultaneously inhibit the production of proinflammatory cytokines and promote the production of anti-inflammatory cytokines [2]. Among the proinflammatory cytokines, interleukin-6 (IL-6) plays the most crucial role in SIRS and the immune defense system [3]. Surgical stress promotes the release of tumor necrosis factor-alpha (TNF-α), derived from monocytes, macrophages, and lymphocytes, along with cytokines such as IL-1 and IL-6. This activation is part of a cascade of inflammatory responses initiated by the hypothalamic-pituitary-adrenal axis [2]. Furthermore, anti-inflammatory responses are initiated by the secretion of interleukin-10 (IL-10) [4], which functions as an anti-inflammatory cytokine by inhibiting monocytes that secrete proinflammatory cytokines, such as TNF-α, IL-6, and interleukin-8 [5].

Following the discovery that the use of most anesthetic agents for general anesthesia or sedation affects the immune system, many studies have investigated these effects [2]. A study on propofol, one of the most widely used intravenous (IV) anesthetic agents, in healthy individuals showed a reduction in both proinflammatory (IL-6) and anti-inflammatory (IL-10) cytokines. However, propofol suppresses the production of the proinflammatory cytokine IL-6 to a greater extent than the anti-inflammatory cytokine IL-10, which may contribute to attenuation of the SIRS [3]. A rodent study showed that propofol decreases TNF-α, IL-1, IL-6, and IL-10 production [6]. In contrast, another study that investigated the anti-inflammatory effects of propofol in patients who underwent craniotomy showed a reduction of the IL-6/IL-10 ratio [7].

Benzodiazepines are widely used IV anesthetic agents known to affect certain aspects of immune function [8]. Midazolam has anti-inflammatory effects that it mediates via its effect on macrophages to inhibit the secretion of proinflammatory cytokines, such as IL-1, IL-6, and TNF-α [8]. In patients who underwent coronary artery bypass grafting surgery, midazolam with propofol decreased IL-6 production [9]. In an in vitro study, IL-10 expression was inhibited by midazolam [10].

Intraoperative hemodynamic variables may also be influenced by the type of anesthetic agent used. A study found a dose-dependent reduction in systolic blood pressure (SBP) and diastolic blood pressure (DBP) with propofol, whereas midazolam did not affect BP [11]. Remimazolam is a new ultra-short-acting, high-affinity gamma-aminobutyric acid-a (GABAa) receptor agonist. Remimazolam is characterized by a pharmacokinetic/pharmacodynamic profile of rapid onset and fast recovery [12]. Notably, its antagonist, flumazenil, confers a significant advantage over propofol. However, there is no current information about the effects of remimazolam on the immune system.

The primary objective of this study was to compare perioperative changes in IL-6 levels between patients receiving remimazolam and propofol during total intravenous anesthesia (TIVA). Secondary objectives included comparisons of IL-10 levels, IL-6/IL-10 ratio changes, intraoperative hemodynamic variables, BIS values, and postoperative opioid requirements. Because TNF-α levels were below the detectable range of the assay, meaningful statistical analysis of TNF-α could not be performed. Furthermore, we explored the perioperative characteristics of remimazolam compared with propofol, one of the most widely used IV anesthetic agents.

## Materials and methods

The study followed a prospective, randomized, assessor-blinded design and was conducted after obtaining approval from the institutional ethics committee of our hospital (approval number: IRB DFH21ORIO415). The study was registered at https://cris.nih.go.kr (KCT0007611) on August 5, 2022. All participants provided written consent before their enrolment in the study. The study was performed in accordance with internationally accepted ethical guidelines, including the 2013 Declaration of Helsinki.

Patient characteristics

The study recruited patients aged between 20 and 65 years with an ASA physical status of I or II who were planned for general anesthesia for total laparoscopic hysterectomy or laparoscopic myomectomy during the period from October 15, 2021, to April 30, 2022.

Exclusion criteria

Subjects were deemed ineligible if they fulfilled any of the following exclusion criteria: an ASA score ≥ III; immune disorders or major systemic disorders that could affect inflammatory cytokine levels; drug allergies; a history of drug or alcohol abuse; existing neuropsychiatric disorders; thyroid diseases; or a body mass index (BMI) >25/m². Additionally, patients were terminated from the study if they were treated with atropine due to bradycardia (heart rate <50 beats) during anesthesia, experienced hypotension (mean arterial blood pressure ≤45 mmHg), and were treated with inotropes, or had SpO₂ levels <90% due to bronchoconstriction.

Statistical analysis

Sample size estimation was performed using G*Power software (version 3.1.9.4; Heinrich Heine University Düsseldorf, Germany) with an F test, assuming a significance level of 0.05 and a power of 80%, based on IL-6 levels measured in a pilot study. Pilot data indicated mean IL-6 values of 25 pg/mL in the remimazolam group and 17 pg/mL in the propofol group, with a standard deviation of 11.2. Based on an effect size of 0.357, a total of 42 participants was required. To account for an anticipated 10% dropout rate, the sample size was increased to 23 subjects per group. The primary endpoint of this study was the perioperative change in IL-6 levels between the remimazolam and propofol groups. Secondary outcomes included IL-10 changes, IL-6/IL-10 ratio changes, intraoperative hemodynamic variables, BIS values, and postoperative opioid requirements.

Statistical analyses were performed using IBM SPSS Statistics for Windows, Version 21 (Released 2012; IBM Corp., Armonk, New York, United States). The Welch t-test was used for intergroup comparisons of age, weight, height, BMI, anesthesia time, operation time, remifentanil dose, nicardipine dose, bispectral index (BIS), heart rate, blood pressure, and the fentanyl and oxycodone doses in the post-anesthesia care unit (PACU).

ASA physical status was compared between groups using Fisher’s exact test. Intergroup differences in IL-6 and IL-10 levels and the IL-6/IL-10 ratio were examined using repeated-measures analysis of variance with Bonferroni-adjusted multiple comparisons. TNF-α was not included in the statistical analysis because its levels were below the detectable range of the assay. Patients with missing cytokine samples were excluded only from the corresponding analysis, and no imputation methods were applied because only one sample was missing.

Intervention

Participants were randomly assigned to either the remimazolam or propofol group using a computer-generated randomization sequence and sequentially numbered, sealed opaque envelopes prepared by an independent investigator not involved in patient management or outcome assessment. Because of the nature of the anesthetic interventions, anesthesiologist blinding was not feasible; however, laboratory personnel and outcome assessors remained blinded to group allocation throughout the study. Patients received intramuscular glycopyrrolate 0.2 mg and IV famotidine 20 mg as premedication 30 minutes prior to surgery. Upon arrival in the operating room, a 22-gauge IV catheter was inserted into the forearm vein, and a baseline blood sample was collected.

Anesthesia was induced using TIVA. Patients in the propofol group received target-controlled infusion (TCI) of propofol (2.5-4 μg/mL) with remifentanil (2 ng/mL), whereas patients in the remimazolam group received manually controlled remimazolam infusion (induction dose: 6 mg/kg/h followed by a maintenance dose of 1-2 mg/kg/h) with remifentanil (2 ng/mL) while monitoring neuromuscular function using train-of-four stimulation. Marsh (Propofol) and Minto (Remifentanil) pharmacokinetic models were applied for the TCI model. Upon loss of consciousness, IV rocuronium 0.6 mg/kg was administered to induce muscle relaxation.

To maintain a BIS of 40-60 for both the P and R groups, the target effect-site concentration (Ce) values of propofol were adjusted within a range of 2.5-4 μg/ml. The maintenance infusion rate of remimazolam after induction was set to 1-2 mg/kg/h, with continuous monitoring. During anesthesia, a tidal volume of 6-8 ml/kg was maintained, with positive end-expiratory pressure set at 5 cm H2O, end-tidal carbon dioxide (ETCO2) at 35-45 mmHg, oxygen saturation maintained above 95%, and peak airway pressure kept below 30 cmH2O. Intraoperative body temperature was maintained using forced-air warming devices in accordance with institutional standard practice. The intraoperative mean blood pressure (MBP) was sustained within 30% of the baseline.

If hypotension or hypertension persisted for five minutes or longer, phenylephrine (100 μg/ml) or nicardipine (1 mg/ml) was administered.

To induce the emergence of patients postoperatively, propofol-, remimazolam-, and remifentanil-TCI were stopped, and IV sugammadex was administered for muscle relaxation. If BIS remained at 60 or lower for 10 minutes after TCI termination in the R group, flumazenil 0.25 mg was administered to conclude anesthesia safely.

Upon complete recovery of consciousness and stable spontaneous breathing, patients were transferred to the PACU after extubation. In the PACU, IV fentanyl 2 μg/kg was administered for analgesia. Additional fentanyl or oxycodone was administered in the PACU according to institutional postoperative pain protocols when patients reported moderate-to-severe pain or numerical rating scale pain scores ≥4. Patients with a post-anesthesia recovery score ≥9 points were transferred to the general ward.

To quantify the serum IL-6, IL-10, and TNF-α levels, venous blood samples were collected at four time points as follows: before anesthesia (T0: baseline), at the end of surgery (T1), 1.5 hours after surgery (T2), and 24 hours after surgery (T3). Each blood sample was immediately centrifuged at 1,000 g after collection. The centrifuged serum samples were stored in a freezer (−20 °C) until assay using commercial kits: Human IL-6 Quantikine ELISA Kit, Human IL-10 Quantikine ELISA Kit, and Human TNF-alpha Quantikine ELISA Kit (R&D Systems, Inc., 614 McKinley Place NE, Minneapolis, MN 55413). All samples were analyzed in duplicate, and the mean value of the duplicate measurements was used for statistical analysis. The minimum detectable concentrations were 0.626 pg/mL for IL-6 (Human IL-6 Quantikine ELISA Kit, R&D Systems, Catalog # S6050), 3.9 pg/mL for IL-10 (Human IL-10 Quantikine ELISA Kit, R&D Systems, Catalog # S1000B), and 6.23 pg/mL for TNF-α (Human TNF-α Quantikine ELISA Kit, R&D Systems, Catalog # STA00D). Intra-assay coefficients of variation (CVs) for IL-6 were 9.7% at T1, 5.4% at T2, and 5.6% at T3, indicating acceptable assay reproducibility during the perioperative period. The markedly elevated CV at T0 (293.2%) was attributed to baseline IL-6 concentrations near the lower detection limit of the assay, which can artificially inflate CV values at extremely low analyte concentrations. Serum samples were aliquoted into 500 μL fractions immediately after separation and stored at −20 °C to minimize repeated freeze-thaw cycles. Each aliquot was thawed only once at the time of analysis. Because of the large number of samples, assays were performed in multiple batches, and samples were randomly distributed across batches to minimize systematic bias. Blood samples were collected between December 8, 2021, and June 17, 2022. Serum samples were separated from SST tubes 30 minutes after collection and stored at −20 °C until analysis. ELISA assays were performed between June and July 2022 after completion of final sample collection.

Intraoperatively, IV crystalloids (plasma solution A) were administered according to the “4-2-1” rule and perioperative fluid guidelines: 4 ml/kg/h for the first 10 kg, 2 ml/kg/h for the second 10 kg, and 1 ml/kg/h for every kg above 20+1 ml/kg/h.

## Results

**Figure 1:**
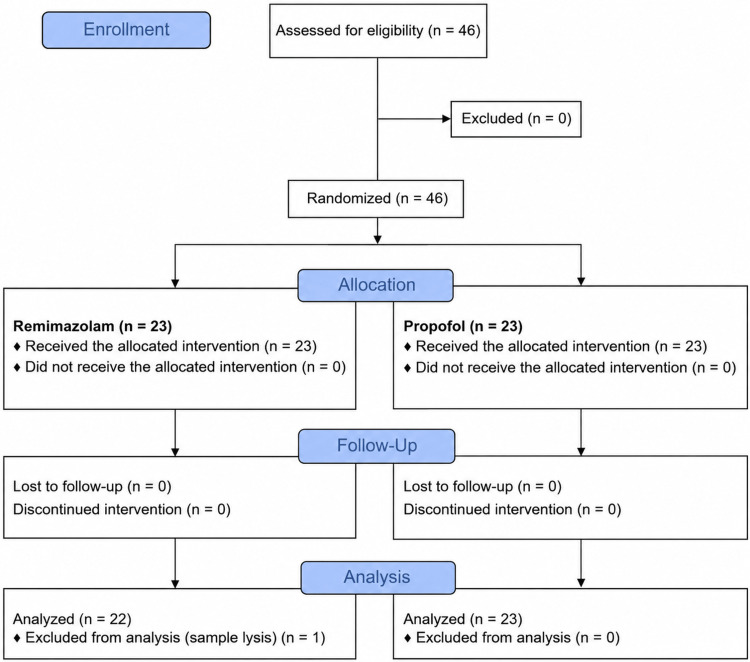
Consolidated Standards of Reporting Trials (CONSORT) flow diagram of patient selection

Table 1 summarizes the characteristics of each patient. Table 2 summarizes the intraoperative opioid requirements, clinical characteristics, and hemodynamic parameters in the remimazolam and propofol groups. The study initially included 46 patients; however, outcome analysis was conducted for 45 patients (22 and 23 in the R and P groups, respectively) due to the loss of one sample in the R group (Figure 1). No patients were excluded after randomization because of bradycardia, hypotension, or desaturation during anesthesia. There was no significant intergroup difference in age, weight, BMI, operation time, and anesthesia duration.

**Table 1:** Demographic and clinical characteristics of all the participants Data are the mean ± SD or number of patients (n). ASA: American Society of Anesthesiologists; SD: standard deviation

	Remimazolam (n = 22)	Propofol (n = 23)	Test statistic	P-value
Age (years)	45.1 ± 4.4	44.1 ± 9.1	t = 0.49	0.625
Height (cm)	159.7 ± 6.2	158.5 ± 4.8	t = 0.67	0.503
Weight (kg)	55.2 ± 6.8	56.2 ± 4.1	t = -0.59	0.555
ASA (I / II)	16 / 6	15 / 8	χ² = 0.10	0.749
BMI	21.6 ± 1.9	22.4 ± 2.1	t = -1.35	0.182

**Table 2:** Intraoperative opioid requirements, clinical characteristics, and hemodynamic parameters in the remimazolam (R) and propofol (P) groups Data are the mean ± SD.  SD: standard deviation; *P < 0.05 SBP: Systolic blood pressure; MBP: mean blood pressure; DBP: diastolic blood pressure; BIS: bispectral index

	R (mean±SD)	P (mean±SD)	Mean Difference (95% CI)	t-value	p-value
Op time	76.62 ± 19.50	73.87 ± 23.20	2.75 (-10.12 to 15.62)	0.427	0.672
Anesthesia time	117.38 ± 21.22	113.70 ± 24.29	3.68 (-10.02 to 17.38)	0.537	0.594
Fentanyl use	0.95 ± 0.22	0.87 ± 0.34	0.08 (-0.09 to 0.25)	0.961	0.343
Fentanyl dose	80.95 ± 29.48	82.61 ± 35.70	-1.66 (-21.32 to 18.00)	-0.168	0.867
Oxycodone use	0.29 ± 0.46	0.57 ± 0.51	-0.28 (-0.57 to 0.01)	-1.912	0.063
Oxycodone dose	1.10 ± 1.81	2.83 ± 2.95	-1.73 (-3.20 to -0.26)	-2.367	0.023
Opioid use	1.90 ± 0.89	2.39 ± 1.27	-0.49 (-1.15 to 0.17)	-1.482	0.146
SBP	124.75 ± 11.99	115.35 ± 12.25	9.40 (2.11 to 16.69)	2.571	0.014
DBP	83.77 ± 7.94	77.34 ± 9.21	6.43 (1.27 to 11.59)	2.485	0.017
MBP	96.95 ± 9.18	89.57 ± 9.88	7.38 (1.65 to 13.11)	2.571	0.014
HR	79.33 ± 9.07	68.41 ± 8.17	10.92 (5.72 to 16.12)	4.184	<0.001
BIS	60.17 ± 5.74	44.01 ± 5.97	16.16 (12.64 to 19.68)	9.151	<0.001

However, the mean intraoperative BIS was significantly higher (t = 9.15, P < 0.001) in the R group (60.17 ± 5.74) compared to the P group (44.01 ± 5.97). Similarly, the mean heart rate was significantly higher (t = 4.184, P < 0.001) in the R group (79.33 ± 9.07 bpm) compared to the P group (68.41 ± 8.17 bpm).

Significantly higher mean SBP, DBP, and MBP were observed in the R group when compared with the P group (SBP: 124.75 ± 11.99 vs. 115.35 ± 12.25, t = 2.571, P = 0.014; DBP: 83.77 ± 7.94 vs. 77.34 ± 9.21, t = 2.49, P = 0.017; MBP: 96.95 ± 9.18 vs. 89.57 ± 9.88, t = 2.571, P = 0.014, respectively). No significant intergroup difference was observed in antihypertensive agent requirements during anesthesia.

There were no significant differences between the two groups in operation time (76.62 ± 19.50 vs. 73.87 ± 23.20 min, t = 0.427, P = 0.672), anesthesia time (117.38 ± 21.22 vs. 113.70 ± 24.29 min, t = 0.537, P = 0.594), fentanyl use (0.95 ± 0.22 vs. 0.87 ± 0.34, t = 0.96, P = 0.343), fentanyl dose (80.95 ± 29.48 vs. 82.61 ± 35.70 μg, t = -0.168, P = 0.867), or total opioid use (1.90 ± 0.89 vs. 2.39 ± 1.27, t = -1.482, P = 0.146).

The mean oxycodone dose was significantly higher in the P group (2.83 ± 2.95 mg) compared to the R group (1.10 ± 1.81 mg) (t = -2.367, P = 0.023), whereas oxycodone use showed a trend toward significance (0.29 ± 0.46 vs. 0.57 ± 0.51, t = -1.912, P = 0.063).

**Figure 2:**
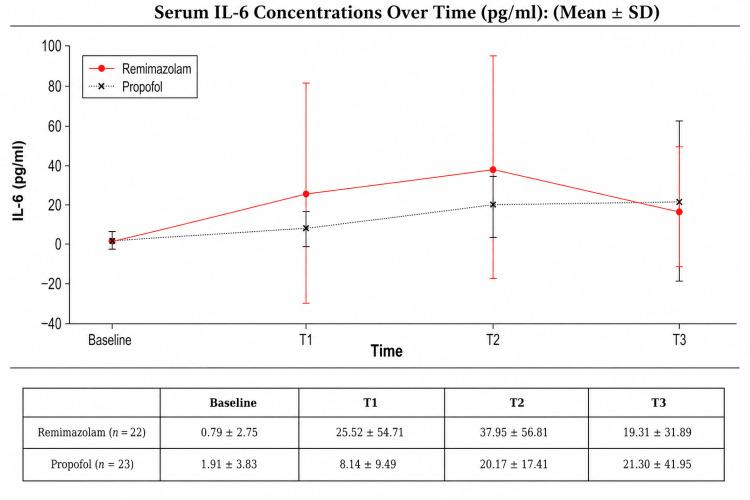
Comparison of IL-6 levels at specific time points between the remimazolam and propofol groups T0 = before anesthetics, T1 = end of surgery, T2 = at 1.5 hours after surgery, T3 = at 24 hours after surgery. P < 0.05 compared with T0 within the group. P > 0.05 between the two groups. A repeated-measures ANOVA with Greenhouse–Geisser correction showed that the interaction between time and group was not statistically significant (F(2.10, 90.08) = 2.43, P = 0.092). IL-6: Interleukin-6

In both groups, the IL-6 levels measured at T1, T2, and T3 were significantly elevated compared to the baseline at T0. In the R group, the IL-6 level at T3 significantly decreased compared to the level at T2, whereas no difference in the levels at the two time points was observed in the P group. Overall, changes over time were confirmed in both groups (P < 0.001). Changes in IL-6 levels did not differ significantly between the two groups (F(2.10, 90.08) = 2.43, P = 0.092) (Figure 2).

**Figure 3:**
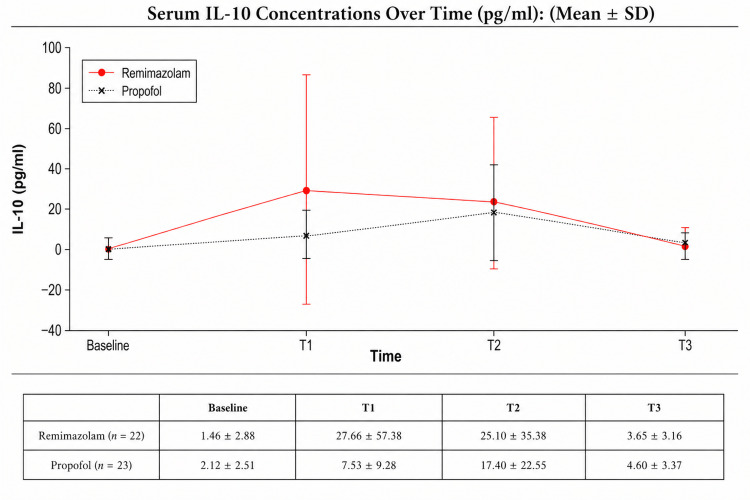
Comparison of IL-10 levels at specific timepoints between the remimazolam and propofol groups T0 = before anesthetics, T1 = end of surgery, T2 = at 1.5 hours after surgery, T3 = at 24 hours after surgery. P < 0.05 compared with T0 for intragroup differences. P > 0.05 for differences between the two groups. A Greenhouse–Geisser correction was applied, and the interaction effect between time and group was not statistically significant, F(1.277, 54.905) = 2.439, P = 0.117. IL-10: Interleukin-10

Upon initiation of surgery, both groups demonstrated a significant increase in IL-10 levels, which reached peak values at T2 (1.5 hours after surgery). Subsequently, a decrease in IL-10 levels at T3 was observed in both groups. Although IL-10 levels changed over time within both groups, there was no statistically significant interaction between time and group (F(1.277, 54.905) = 2.44, P = 0.117) (Figure 3).

**Figure 4:**
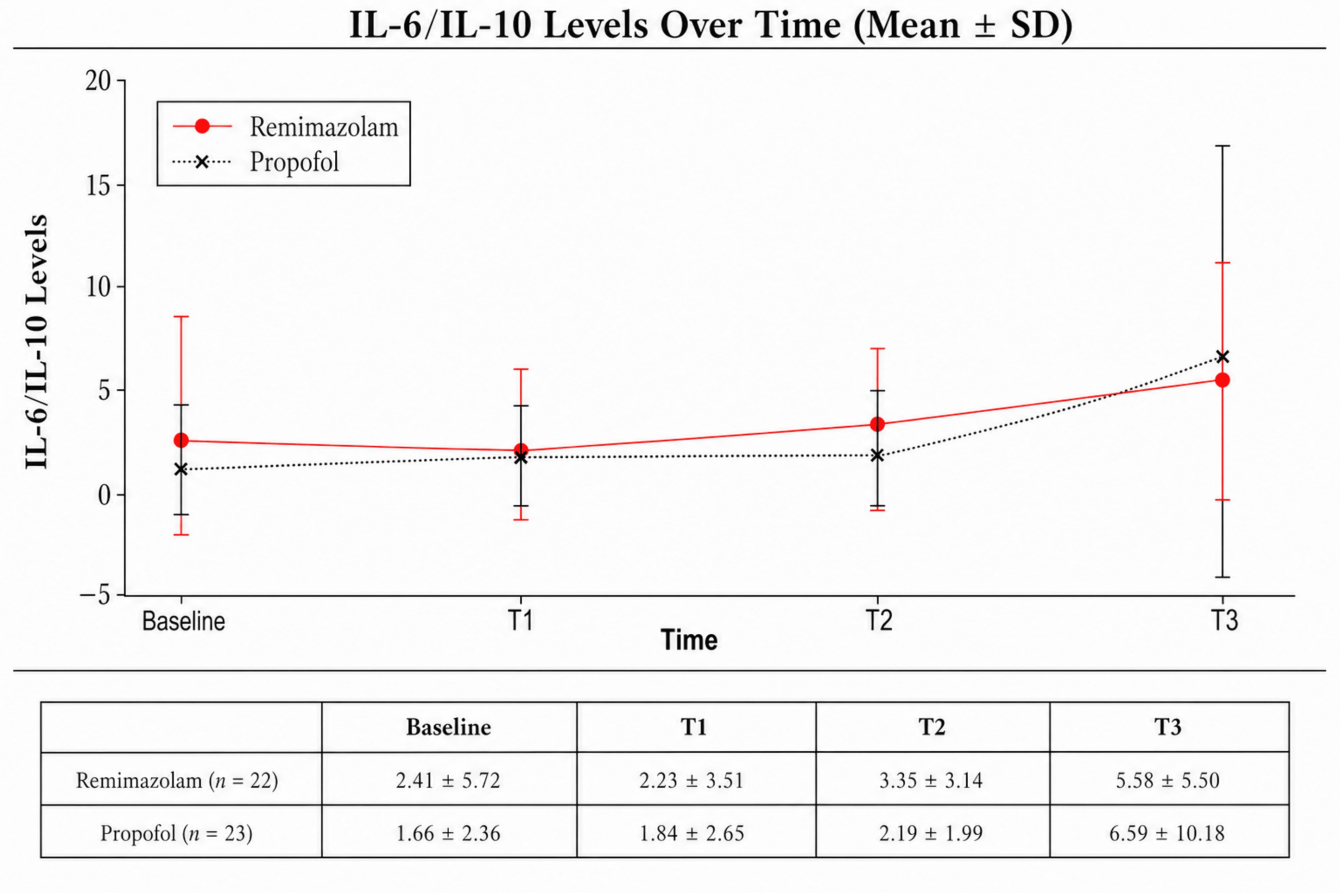
Comparison of IL-6/IL-10 levels at specific timepoints between the remimazolam and propofol groups T0 = before anesthetics, T1 = end of surgery, T2 = at 1.5 hours after surgery, T3 = at 24 hours after surgery. P < 0.05 compared with T0 within the group. P > 0.05 for differences between the two groups. A Greenhouse–Geisser correction was applied, and the interaction effect between time and group was not statistically significant, F(1.874, 80.586) = 0.461, P = 0.620 IL-6: Interleukin-6; IL-10: Interleukin-10

Statistically significant changes in the IL-6/IL-10 ratio over time were observed in both groups, with an increase over time. However, there was no significant intergroup difference in the change over time (F(1.874, 80.586) = 0.46, P = 0.620) (Figure 4).

TNF-α levels were below the detectable range of the assay, precluding meaningful analysis.

## Discussion

This study investigated the impact of remimazolam, a novel, ultra-short-acting benzodiazepine, on the levels of inflammatory cytokines and its influence on hemodynamic parameters compared to propofol during general anesthesia. Earlier research examining the impacts of propofol and midazolam on inflammatory cytokines provided some insight into their effects on immune function. However, propofol is recognized for its superior ability to inhibit inflammation [13]. In this study, we hypothesized that remimazolam would demonstrate perioperative inflammatory cytokine patterns that could be compared with those observed with propofol, based on its pharmacological similarity to midazolam. While IL-6 showed significant changes over time, IL-10 levels also demonstrated significant temporal changes within both groups; however, no significant interaction between time and group was observed. The IL-6/IL-10 ratio increased postoperatively and peaked at T3 without significant intergroup timepoint-based differences. This increase may reflect a pro-inflammatory shift following surgical stress rather than an anesthetic-specific effect. These findings suggest that no statistically significant intergroup differences were observed in perioperative inflammatory cytokine patterns.

IV anesthetics in general anesthesia impact various parameters, including blood pressure, heart rate, BIS, and inflammatory cytokines. Remimazolam, a benzodiazepine akin to midazolam, has been reported to exhibit sedative effects similar to midazolam and clinical efficacy comparable to propofol [12,14]. Favorable hemodynamic profiles, consistent with midazolam's known characteristics [11], have been reported during anesthesia. Although the remimazolam group demonstrated significantly higher intraoperative blood pressure and heart rate values, these findings should be interpreted cautiously. Because BIS values were consistently higher in the remimazolam group despite attempts to maintain comparable anesthetic depth, the observed hemodynamic differences may reflect inadequate equivalence of hypnotic depth rather than intrinsic cardiovascular stability associated with remimazolam itself. In addition, BIS monitoring may underestimate hypnotic effects during benzodiazepine-based anesthesia, further complicating the interpretation of the hemodynamic findings. No significant difference was observed between the two groups in the requirement for antihypertensive agents.

Furthermore, no significant difference in intraoperative opioid use was observed; therefore, differences in postoperative opioid requirements should be interpreted cautiously. A previous study highlighted the unintended consequences of reducing intraoperative opioids, leading to worsened postoperative pain and increased opioid use [15]. Similarly, no difference in fentanyl requirements was observed in the recovery room, whereas oxycodone requirements were significantly higher in the propofol group. Although postoperative oxycodone requirements differed between groups, interpretation of this finding is limited because detailed postoperative pain assessments were not performed. Although BIS values were targeted between 40 and 60, persistently higher BIS values in the remimazolam group may reflect limitations of BIS monitoring during benzodiazepine-based anesthesia and should be considered when interpreting the cytokine and hemodynamic findings. Because this study was exploratory and not powered for repeated-measures effect-size estimation across multiple secondary outcomes, estimated marginal means and formal effect-size analyses were not performed and should be incorporated in future adequately powered studies.

Given the lack of a TCI model for remimazolam, weight-based dosing was employed while restricting the dose to 2 mg/kg/h even with high BIS values (50-60). BIS values during anesthesia were higher in the R group, consistent with midazolam's reported average BIS values [16]. In contrast to intraoperative awareness, there were no instances of awareness among patients who received remimazolam in the PACU. However, considering potential limitations, an objective tool such as the Auditory Evoked Potential Index (AEPindex) could confirm accurate anesthetic depth with remimazolam [16].

The study involved relatively healthy patients with ASA physical status I or II who were 20 to 65 years old; therefore, the findings may not be generalizable to older patients or those with significant comorbidities. TNF-α levels were below the detectable range of the assay, preventing meaningful analysis. Because TNF-α often demonstrates an earlier and shorter perioperative peak compared with IL-6, the selected sampling schedule may have reduced the likelihood of detecting clinically meaningful TNF-α changes. In addition, both IL-6 and IL-10 showed larger standard deviations than anticipated in the pilot study, and statistical significance may have been affected by cytokine variability and the relatively small sample size. The markedly elevated baseline intra-assay CV for IL-6 likely reflected very low preoperative cytokine concentrations near the assay detection limit and should therefore be interpreted cautiously. Although no randomized patients were excluded after group allocation, the predefined exclusion criteria may still represent a potential source of attrition bias in future studies. Further studies should include larger and more diverse patient populations, standardize anesthetic depth, and incorporate earlier postoperative sampling time points to better characterize perioperative TNF-α kinetics.

## Conclusions

In conclusion, no significant intergroup differences were observed in perioperative IL-6 and IL-10 responses between remimazolam and propofol. However, because anesthetic depth was not fully equivalent between groups, as reflected by higher BIS values in the remimazolam group, the observed cytokine and hemodynamic findings should be interpreted cautiously. The observed differences in intraoperative blood pressure and heart rate may therefore reflect lighter anesthesia rather than intrinsic pharmacologic effects of remimazolam. The absence of statistically significant differences should not be interpreted as evidence of immunologic equivalence between the two anesthetic agents. These findings highlight the need for further investigation of remimazolam as an alternative anesthetic agent. Further large-scale studies with standardized anesthetic depth are required to clarify the immunomodulatory and hemodynamic effects of remimazolam in broader surgical populations.
